# Geriatric Traumatic Brain Injury in China

**DOI:** 10.1007/s13670-012-0018-1

**Published:** 2012-06-19

**Authors:** Xianwei Zeng, Shun Pan, Zhenbo Hu

**Affiliations:** Department of Neurosurgery, Affiliated Hospital of Weifang Medical University, 465 Yuhe Road, WeiFang, 261031 Shandong People’s Republic of China

**Keywords:** Traumatic brain injury, Geriatric, Trauma, Injury, Epidemiology, Outcomes

## Abstract

Traumatic brain injury (TBI) is one of the major causes of morbidity and mortality in China. The elderly population has the higher rates of TBI-related hospitalization and death. Traffic accidents are the major cause for TBI in all age groups except in the group of 75 years and older, in which stumbles occurred in nearly half of those who suffered TBI. Older age is known to negatively influence outcome after TBI. To date, investigators have identified a panel of prognostic factors that include initial Glasgow Coma Scale score, comorbidities, cerebrospinal fluid leakage, associated extracranial lesions, and other factors such as cerebral perfusion pressure on recovery after injury. However, these aspects remain understudied in elderly patients with TBI. In the absence of complete clinical data, predicting outcomes and providing good care of the elderly population with TBI remain limited. To address this significant public health issue, a refocusing of research efforts is justified to prevent TBI in this population and to develop unique care strategies for achieving better clinical outcomes of the patients with TBI.

## Introduction

Traumatic brain injury (TBI) is frequently referred to as the silent epidemic because the problems that result from it, such as impaired memory, are often invisible. Within this silent epidemic, there is a seemingly silent population: older adults with TBI. Older age is known as a factor that negatively influences outcome after TBI [1, 2••, 3, 4]. The patient group older than 60 years has the most significant association with unfavorable outcomes; however, studies on the detailed correlations and mechanisms between the age and clinical outcome of patients with TBI are sparse, and development of appropriate clinical cares of elderly patients with TBI are thus limited. Furthermore, despite the fact that investigators have identified many risk factors that may affect the prognosis of geriatric neurotrauma, such as Glasgow Coma Scale (GCS) score, pupillary light reflex, subdural hematoma, and subarachnoid hemorrhage [2••], the prognostic significance of these factors in older adults with TBI remains understudied. The relative neglect of these aspects in neuroscience research may partially explain why predicting outcomes and providing care in the older adult population with TBI remains so problematic. Paying attention to these factors might improve the outcome of the elderly TBI in clinical treatment. This review addresses the epidemiology of TBI in older adults and the factors affecting patient outcomes in China, with focus on the implications of the current state of knowledge and identifying areas for future research and clinical applications.

## Epidemiology

There is limited data to support epidemiologic transition of geriatric TBI in China during the past decade. The situation was mainly due to lack of valid data on vital statistics in China because of a lack of coherent systems for national registration of TBI. Most of current studies on TBI in China are sporadic and retrospective, which may be uninformative and nonconclusive to some extent [5, 6]. However, there has been a large, prospective cohort study involving a group of representative samples to estimate the demographic profiles of TBI patients in eastern China, which is the most economically developed area in China, with a population estimated to be 373.3 million or 28.7 % of the total population of the People’s Republic of China [7••]. The study revealed that epidemiology of TBI in young and physically active people aging from 15 to 54 years old is constituted of 71.5 % of all patients, while the elderly aged 55 years and older account for 19.1 %.

## Mechanisms of Injury

In China, traffic accidents were the major cause for TBI in all age groups except for the group of 75 years and older, in which stumbles occurred in nearly half of those (44.7 %) who suffered from TBI. Stumble was the most common cause of TBI in elderly patients aged 75 years and older, which may be associated with the handicap imposed by age in their daily life of this group. The elderly were more likely to injure themselves, and this had a significant impact on the mortality and morbidity. Earlier studies have showed that elderly persons aged 65 years and older with severe TBI had a higher mortality as compared with their younger counterparts [8]. In the past decade, traffic accidents increased along with the increasing number of vehicles and motor bicycles [9]. Such a pattern was not consistent with other TBI studies in developed countries, in which the second-most common cause of TBI was fall-related injury, which mainly affected the elderly population. However, in the United States, falls are the leading cause of TBI for older adults, and motor vehicle crashes (MVCs) are second. Assaults only account for 1 % of TBIs in older adults, and all other known causes account for 17 %, though more than 21 % of TBI occurred in elderly people aged 65 years and older [10]. In the past 10 years, traffic accidents were the leading cause of TBI in China, which was much higher than the United States and European countries. This is due to the rapidly increasing number of vehicles in this country, while motorcyclists and pedestrians were the most vulnerable group of road users. The patterns of incidence by age and sex are in agreement with previous findings [11, 12].

## Sex

Though few studies have addressed this issue, available data showed that men were associated with a higher incidence of TBI in older adults [13•]. This is partially due to the fact that men are more likely injured as motorcyclists. In addition, studies involving both animals [14] and patients [15] have shown that women are associated with better outcome after TBI. Further study suggested that the better improvement in women is correlated with the level of estrogen and progesterone [16, 17], which contribute to the maintenance of adequate cerebral perfusion. These hormone levels are changed dramatically following menopause in women. However, because other experimental [18] and clinical [19] studies have refuted these findings, the data remain inconclusive at this point. Further studies are needed to illustrate if the effects of these hormones are neuroprotective or coeffective with other factors, which could have implications for therapeutic intervention in clinic for TBI.

## Chronic Health Conditions in Older Adults with TBI

There is growing recognition that TBI is a highly variable and complex systemic disorder that is refractory to therapies that target individual mechanisms. It is even more complex in elderly patients, in whom frailty, previous comorbidities, altered metabolism, and a long history of medication use are likely to complicate the secondary effects of brain trauma. Study found that 73 % of elderly TBI patients had a medical condition before injury, compared with 28 % of younger adults with TBI [20]. This significant increase in comorbidity is an important factor to be considered in providing primary and secondary prevention efforts for this group of patients. The relative risk of fall in older adults with diabetes mellitus is twice that of with without diabetes mellitus [21]. Because falls are the second cause of TBI in older adults, this population may be appropriate to receive unique prevention strategies, and this is obviously an area that needs further study. In addition, study showed that 9 % of elderly patients with TBI were taking warfarin before injury [22], and this was associated with more severe TBI and a higher rate of mortality. The Practice Management Guidelines for Geriatric Trauma indicated that the presence of comorbid conditions adversely affect outcome after an injury, but this effect becomes less pronounced with older patients. Throughout the course of an intensive care unit stay, certain preexisting conditions place the elderly patients with TBI at greater risk for secondary complications. These complications lengthened the time of hospital stay and increased mortality of patients with TBI. A higher number of medical comorbidities also were found to be associated with longer rehabilitation stays in older adults with TBI. The development of subsequent complications also has been related to severity of injury. Study also found that older TBI patients with preexisting pulmonary disease were more than 1.6 times as likely to develop pneumonia as those without pulmonary disease [23]. Therefore, it has been suggested that the type of preexisting condition, as well as injury severity and mechanism, must be taken into consideration when endeavoring to predict outcomes for older TBI patients [10]. Comorbid dementia or mild cognitive impairment, whether from Alzheimer’s disease or other etiologies, is also a risk factor for TBI [24, 25] and for slower recovery from TBI [26]. Cognitive impairment is underdiagnosed in older patients [26]. Even when diagnosed by care providers experienced with elderly patients with TBI, it is often difficult to distinguish what part of cognitive impairment is due to preexisting dementia and what is due to TBI. Preexisting cognitive impairment confounds the diagnosis of TBI in such patients after trauma. Therefore, further study is needed in this area to better explicate the influence of comorbid conditions on the incidence of and outcomes after TBI in older adults.

## TBI Outcome in Older Adults

Older age has long been recognized as an independent predictor of worse outcome from TBI [1, 2••, 3, 4]. The mechanism by which this occurs remains unknown. Because research was unable to pinpoint useful clinical predictors of lesion formation, head computed tomography (CT) scans are now recommended for all patients aged 65 years and older presenting with neurological symptoms and signs or history of head trauma to aid TBI diagnosis. There was a higher incidence of chronic subdural hematoma and of intracerebral hematoma in this group. Because of this, the incidence of intracranial hematoma in the older adults (9.4 %) was significantly higher than in the younger group (2.7 %) [27]. The costs of treating TBI in older adults are also more expensive [28]. Mortality rates in adults with severe TBI aged 55 years and older are significantly higher than those reported in younger patients [26, 27, 29]. Mortality rates for older patients with mild TBI are also substantially higher than for their younger counterparts [30, 31]. Although a single study has reported that adults aged 60 years and older who suffered mild TBI had significantly better functioning (*P* < 0.05) at 1 month postinjury on the Glasgow Outcome Scale (GOS) than younger patients with mild TBI, significance was not maintained when controlling for employment status [4]. In the studies that examined disability after TBI, there were evidences to suggest that older adult TBI survivors had greater dependence than younger survivors using global outcome measures including the GOS [32] and the Functional Independence Measure [31]. Given that older adults with TBI demonstrate slower functional recovery from TBI, this should be an important factor to be considered in the future intervention and study on the disorder.

## Conclusions

Information on epidemiology of TBI in China is currently incomplete due to lack of available statistical data and coherent systems for national registration of TBI. Government-sponsored programs for nationwide registration of TBI are being established. Traffic accidents are the major cause for TBI in all age groups except for the group of 75 years and older in China. Understanding the mechanism of TBI remains a challenge, with the primary insult occurring at the scene of the injury, after which a cascade of events starts. At this stage, it is difficult to estimate the amount of damage that may take place; however, efforts and attention should be focused on limiting the effects of secondary brain injury. These effects can be minimized through the use of simple techniques such as proper oxygenation, restoration of volume depletion, and preventing hypothermia. Intensive care and anesthesia management is a continuation of care with the aim of minimizing secondary brain damage. With the advancement in our knowledge of pathophysiology about TBI, the use of new agents has helped improve outcomes of TBI. This improvement is expected to continue with a better understanding of the mechanism of damage including apoptosis and factors associated with this disorder, and also may lead to improvement of cognitive dysfunction associated with TBI in elderly patients.
